# Association between the weight-adjusted waist index and age-related macular degeneration in US adults aged≥40 years: the NHANES 2005–2008

**DOI:** 10.3389/fmed.2025.1552978

**Published:** 2025-03-06

**Authors:** Yuting Wu, Yuxin Liu, Ziman Jiao, Xin Chen, Haiyu Li, Yunhao Zhou, Guanghui Liu

**Affiliations:** ^1^Department of Ophthalmology, Affiliated People’s Hospital (Fujian Provincial People’s Hospital), Fujian University of Traditional Chinese Medicine, Fuzhou, China; ^2^Eye Institute of Integrated Chinese and Western Medicine, Fujian University of Traditional Chinese Medicine, Fuzhou, China; ^3^Department of Bioengineering, College of Biological Science and Biotechnology, Fuzhou University, Fuzhou, China

**Keywords:** weight-adjusted waist index, age-related macular degeneration, NHANES, obesity, non-linear association

## Abstract

**Objective:**

The association between the weight-adjusted waist index (WWI) and age-related macular degeneration (AMD) in US adults aged 40 years and older is unknown. The goal of this study was to ascertain a possible association between the two.

**Methods:**

Data were obtained from the National Health and Nutrition Examination Survey (NHANES) in the US from 2005 to 2008. The WWI was calculated by dividing waist circumference (WC) by the square root of body weight (kg). AMD was diagnosed based on distinctive features observed in the fundus, using a standard classification system. Weighted logistic regression analyses were conducted to investigate the association between the WWI and AMD. Spline smoothing and threshold effects were applied to explore non-linear correlations. Subgroup analyses were performed to identify underlying covariates affecting this relationship. In addition, receiver operating characteristic (ROC) curve analysis was used to evaluate the predictive power of the WWI for AMD.

**Results:**

A total of 5,132 participants were enrolled in this study. The results showed a significant positive association between the WWI and risk of AMD (OR = 1.76 (1.52, 2.04); *p* < 0.0001). When the WWI was categorized into tertiles, the highest group exhibited a stronger association compared to the lowest tertile (OR = 2.90 (2.18, 3.86); *p* < 0.0001) in model 1. The subgroup analyses and interaction tests indicated that the relationship between the WWI and AMD was stable across various populations. The spline smoothing and threshold effects showed a positive non-linear correlation between the WWI and AMD incidence. Furthermore, compared to body mass index (BMI), WC, and weight, the WWI showed better predictability for AMD, as shown by the ROC analysis.

**Conclusion:**

There exists a positive non-linear association between the WWI and AMD in US adults aged 40 years and older. The WWI-related obesity management is necessary for the prevention and treatment of AMD.

## 1 Introduction

Age-related macular degeneration (AMD) is one of the leading causes of irreversible blindness worldwide, damaging the macular area of the retina (1). It is classified into neovascular and non-neovascular types (1). Both types can lead to the loss of central vision, even resulting in severe and permanent visual impairment and blindness (2, 3). In addition, AMD is a major contributor to significant visual impairment in individuals over the age of 50 in high-income countries (4). It is estimated that 8.69% of the global population is affected by this disease. By 2020, the number of individuals with AMD was 196 million; by 2040, this prevalence is estimated to reach 288 million (5).

Relevant studies have shown that AMD incidence is closely related to genetic and environmental factors, including aging and cardiovascular diseases (6, 7). A comprehensive study conducted in Taiwan, employing a range of methodologies, identified that ARMS2/HTRA1 risk alleles are instrumental in elucidating the molecular and genetic mechanisms underlying neovascular AMD (8). A systematic review highlighted that genetic predispositions, including variants in CFH, APOE, and pathways related to oxidative stress, significantly influence the risk and progression of AMD (9). In addition, certain genes associated with lipid transport and complement regulation have been implicated in AMD pathogenesis (10). Pharmacological interventions may also impact AMD incidence. For instance, a clinical study demonstrated that metformin significantly reduced the risk of AMD in Taiwanese patients with type 2 diabetes mellitus (11) and also lowered the risk of AMD-related conditions such as dementia, hypertension, and heart failure (12–14). Furthermore, glucagon-like peptide-1 receptor agonists (GLP-1RAs) have been suggested to decrease the risk of exudative AMD (15), while sodium-glucose cotransporter-2 (SGLT2) inhibitors exhibit a robust protective effect against macular degeneration in diabetic patients (16). An experimental study also demonstrated that atorvastatin (AT) confers protection against AMD by inhibiting the progression of pyroptosis (17).

AMD has been independently correlated with a variety of diseases. A European study on ocular diseases found a correlation between hypertension and an increased likelihood of early AMD (18). Furthermore, a retrospective cohort study by Lee et al. (19) reported a heightened incidence of exudative AMD among patients with diabetes mellitus. High levels of plasma high-density lipoprotein cholesterol (HDL-C), which represent dyslipidemia, were causally associated with an increased risk of advanced AMD in European and Asian populations according to a multiethnic study (20). The development of AMD affects individuals’ quality of life and increases socioeconomic burdens (21). Therefore, early prediction and diagnosis of AMD hold significant clinical value.

Obesity is influenced by human metabolism, ethnic heritage, individual behaviors, and environmental changes (22). It is expected to affect more than half of American adults in 29 states by 2030 (23). Previous studies have shown that obesity increases the risk of several illnesses (24–27). In terms of AMD incidence, it has also been identified as a risk factor (28, 29). Body mass index (BMI) is a commonly used measure for obesity assessment and classification; however, it cannot accurately distinguish between fat, lean mass, and fat distribution in individuals (30). In addition, waist circumference (WC), the waist-to-height ratio (WHtR), and the waist–hip ratio (WHR) are commonly used clinical parameters for assessing central obesity.

A population-based Taiwanese survey reported that WC and the WHtR have similar efficacy and are superior to BMI and the WHR for predicting diseases such as hypertension and dyslipidemia (31). However, these indicators cannot quantify fat content and do not accurately reflect overall adiposity or leanness status. Therefore, exploring novel indices that better represent obesity is necessary.

Recognized as a new assessment tool for obesity, the weight-adjusted waist index (WWI) can better distinguish between fat distribution and muscle mass within the body. In terms of ocular diseases, the WWI has become a vital parameter (32). While the WWI has a strong predictive utility for other diseases (33–35), the link between the WWI and AMD has not been investigated.

In this study, the National Health and Nutrition Examination Survey (NHANES) database from 2005 to 2008 was utilized to investigate the association between the WWI and AMD.

## 2 Methods

### 2.1 Study population

**Figure 1:**
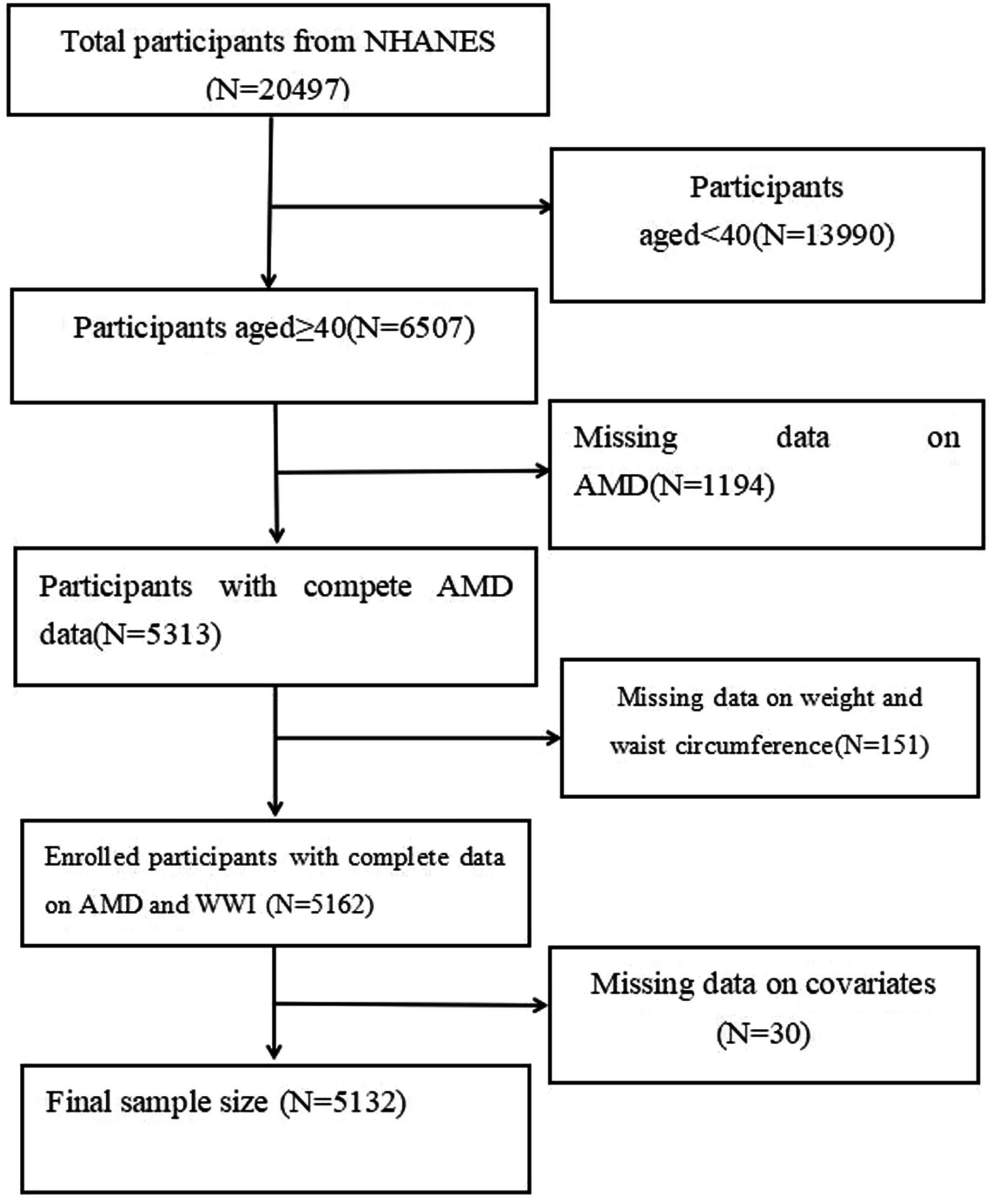
Flowchart of the sample selection from the NHANES 2005–2008 cycles.

The NHANES database uses a complex, multistage probability sampling design to represent the diet and health conditions of the non-institutionalized civilian population in the United States. It ensures a representative sample of the total U.S. population through random selection, under the administration of the Centers for Disease Control and Prevention. The National Center for Health Statistics (NCHS) initially collected the data. All data calculations required the use of sample weights, in accordance with the NCHS analysis guidelines. Approximately 5,000 American participants were tested each year. In this study, the NHANES 2005–2006 and 2007–2008 study cycles were included. A total of 20,497 individuals were initially included. All survey participants provided written informed consent before the survey. The exclusion criteria included individuals under the age of 40 years (*n* = 13,990), individuals with missing data on weight and waist circumference (*n* = 151), and individuals with missing data on other covariates (*n* = 30). In the end, 5,132 participants were included (Figure 1).

### 2.2 Exposure variable

The WWI was used as the exposure variable in the study. It is a novel obesity index evaluating an individual’s central obesity degree based on waist circumference and weight measurements, both of which were taken at a designated mobile examination center (MEC) where experimental measurements were conducted under controlled conditions (36, 37). The WWI was calculated as follows (32):


$$WWI=WC\;\left(\mathrm{cm}\right)÷\surd \left(\mathrm{weight}\right)\left(\mathrm{kg}\right)$$

### 2.3 Outcome variables

The participants’ AMD data were assessed from the NHANES examination dataset. The diagnosis was based on accurate fundus photography. Individuals aged 40 and above throughout the NHANES cycles from 2005 to 2008 underwent retinal photography. A Canon CR6 non-mydriatic camera, equipped with a Canon 10D camera on the back, was used to capture two 45-degree non-mydriatic fundus photographs of the retina in each eye of every survey participant. At the University of Wisconsin–Madison in America, each retinal image of the participants was evaluated and scored by experienced experts using a taxonomy standard based on a modified version of the Wisconsin age-related macular disease grading classification system (38). On the NHANES website, a detailed retinal image processing manual was available (39). At least two specialists rated the scores, and if inconsistencies arose, a third researcher would intervene to resolve them. Early AMD was defined by the presence of drusen with a grid area larger than a 500-μm circle and/or pigmentary abnormalities, while late AMD was characterized by the appearance of geographic atrophy or exudative AMD (including subretinal detachments, subretinal hemorrhage, visible subretinal new vessels, subretinal fibrous scars, or laser treatment scars for AMD) (40, 41). If the participants had retinal images of both eyes, valid analyses were performed selectively on the retina of the worse eye.

### 2.4 Covariates

The covariates considered in the study included sex (male and female), age (years), ethnicity (Mexican American, other Hispanic, non-Hispanic white, non-Hispanic Black, and other ethnicities), education level (less than 9th Grade, 9-11th grade, high school grad/GED or equivalent, some college or associate degree, and college graduate or above), marital status (married, widowed, divorced, separated, never married, living with partner), poverty–income ratio (PIR), waist circumference (WC), weight, body mass index (BMI), smoking status (yes/no), hypertension status (yes/no), and diabetes status (yes/no/borderline). BMI was calculated as body weight (kg) divided by height (m)^2^ at the MEC. Smoking status was determined by whether the participants had smoked at least 100 cigarettes in their lifetime. Hypertension was defined based on the question: “Were you told by a doctor or other health expert that you have hypertension, also called high blood pressure?” The participants who answered “Yes” to the question, “Has your doctor told you that you have diabetes?” were characterized as having diabetes (excluding gestational diabetes) (32). All details regarding these covariates were accessed directly from the NHANES website.

### 2.5 Statistical analysis

The continuous covariates were presented as mean ± standard deviation (SD), while the categorical covariates were reported as percentages. For the continuous covariates, *p*-values were calculated using weighted linear regression analysis. For the categorical covariates, weighted chi-square tests were used to assess the data.

Weighted multiple logistic regression analysis was performed. This analysis was divided into three models. Model 1 was established without any adjustments. Model 2 was adjusted for sex, age, and ethnicity in terms of the demographic variables. Model 3 included all the adjustments of Model 2, plus education level, marital status, poverty-income ratio, body mass index (BMI), smoking status (smoked at least 100 cigarettes in life), and diabetes and hypertension status, accounting for a range of potential factors affecting the relationship between the WWI and AMD. To enhance the robustness and reliability of the analytical results, the WWI was transformed from a continuous variable to a categorical variable, categorized into tertiles through sensitivity analyses. Subgroup analyses and interaction tests were used to further identify variables that influence this association across diverse populations. A potential linear or non-linear correlation was detected using smoothing curve fitting and threshold effect analyses. Data analysis was performed using R^1^ and EmpowerStats (version 4.2). In addition, a *p*-value of <0.05 was considered statistically significant.

## 3 Results

### 3.1 Baseline characteristics of the study population

A total of 5,132 individuals were included in this study, with 50.16% being female and 49.84% being male. Among them, 391 participants had AMD, representing 7.62% of the total sample. The WWI tertiles were categorized as Tertile 1 (8.59–10.85), Tertile 2 (10.85–11.52), and Tertile 3 (11.52–15.70). As shown in Table 1, statistically significant differences between the WWI tertiles were found for sex, age, ethnicity, education level, marital status, PIR, waist circumference, weight, BMI, diabetes status, hypertension status, smoking status, and AMD (all *p* < 0.05). The highest WWI tertile group tended to consist of older female individuals with lower educational attainment and PIR compared to those in Tertile 1. In addition, the participants in the higher WWI groups were more likely to be non-Hispanic white and widowed, considering ethnicity and marital status. Higher proportions of waist circumference, weight, and BMI were related to higher WWI tertiles. There was a higher likelihood of experiencing diabetes, hypertension, and AMD and having smoked at least 100 cigarettes in a lifetime in the higher WWI tertiles (all *p* < 0.05). In addition, after the inclusion of the WHtR, the relevant characteristics of the study population are shown in Supplementary Table 1, while Supplementary Tables 4, 5 show the population characteristics by sex.

**Table 1:** Baseline characteristics of the study population.

Weight-adjusted waist index	Tertile 1 *N* = 1,711	Tertile 2 *N* = 1,697	Tertile 3 *N* = 1,724	*P*-value
Sex (%)				<0.0001
Male	48.14 (44.82, 51.47)	51.37 (48.35, 54.39)	40.42 (38.21, 42.67)	
Female	51.86 (48.53, 55.18)	48.63 (45.61, 51.65)	59.58 (57.33, 61.79)	
Age (years)	52.04 (51.26, 52.83)	56.18 (55.34, 57.02)	62.25 (61.30, 63.21)	<0.0001
Ethnicity (%)				<0.0001
Mexican American	2.81 (2.12, 3.71)	5.17 (3.71, 7.16)	6.09 (3.95, 9.29)	
Other Hispanic	2.48 (1.50,4.09)	2.43 (1.56, 3.78)	4.27 (2.84, 6.39)	
Non-Hispanic white	77.73 (73.45, 81.49)	78.67 (73.95, 82.73)	77.96 (72.38, 82.69)	
Non-Hispanic Black	12.41 (9.55, 15.96)	8.32 (6.11, 11.23)	7.54 (5.53, 10.21)	
Other ethnicities	4.58 (3.04, 6.83)	5.41 (3.98, 7.33)	4.12 (2.71, 6.22)	
Education level (%)				<0.0001
Less than 9th Grade	3.27 (2.32, 4.60)	6.05 (4.35, 8.38)	10.13 (8.06, 12.65)	
9-11th grade	9.11 (7.19, 11.47)	10.20 (8.02, 12.90)	14.63 (12.56, 16.96)	
High school grad/GED or equivalent	22.43 (20.07, 24.97)	28.85 (25.63, 32.29)	29.49 (27.26, 31.82)	
Some college or associate degree	29.38 (26.52, 32.43)	28.27 (24.79, 32.04)	26.98 (23.17, 31.17)	
College graduate or above	35.81 (31.42, 40.45)	26.62 (22.88, 30.74)	18.78 (15.68, 22.32)	
Marital status (%)				<0.0001
Married	65.80 (62.00, 69.41)	68.37 (64.56, 71.95)	60.76 (58.01, 63.44)	
Widowed	4.03 (3.08, 5.26)	7.53 (6.28, 9.00)	14.46 (12.26, 16.97)	
Divorced	15.58 (13.08, 18.46)	12.80 (10.65, 15.31)	13.20 (10.90, 15.89)	
Separated	2.37 (1.54, 3.62)	3.04 (2.07, 4.44)	1.60 (1.11, 2.31)	
Never married	7.01 (5.31, 9.20)	4.83 (3.63, 6.39)	6.06 (4.87, 7.53)	
Living with partner	5.21 (4.16, 6.51)	3.44 (2.42, 4.86)	3.92 (2.66, 5.73)	
PIR	3.62 (3.48, 3.75)	3.38 (3.22, 3.53)	2.90 (2.75, 3.04)	<0.0001
Waist circumference (cm)	90.02 (89.22,90.82)	102.58 (101.90,103.26)	112.44 (111.47,113.42)	<0.0001
Weight (kg)	76.70 (75.42, 77.98)	85.27 (84.15, 86.38)	89.08 (87.56, 90.60)	<0.0001
BMI (kg/m^2^)	26.14 (25.78, 26.51)	29.56 (29.27, 29.85)	32.59 (32.11, 33.06)	<0.0001
Diabetes (%)				<0.0001
Yes	3.52 (2.80, 4.42)	9.53 (7.35, 12.26)	20.91 (18.49, 23.56)	
No	95.55 (94.29, 96.53)	88.92 (85.89, 91.37)	75.48 (72.45, 78.27)	
Borderline	0.93 (0.51,1.69)	1.55 (0.92, 2.60)	3.61 (2.59, 5.00)	
Smoking (%)				0.0004
Yes	47.25 (43.63, 50.91)	53.50 (49.84, 57.13)	55.18 (52.25, 58.07)	
No	52.75 (49.09, 56.37)	46.50 (42.87, 50.16)	44.82 (41.93, 47.75)	
Hypertension (%)				<0.0001
Yes	28.71 (25.93, 31.66)	43.10 (39.42, 46.85)	57.42 (54.58, 60.20)	
No	71.29 (68.34, 74.07)	56.90 (53.15,60.58)	42.58 (39.80, 45.42)	
AMD (%)				<0.0001
No	96.14 (94.81, 97.15)	94.16 (92.31, 95.58)	89.57 (87.59, 91.27)	
Yes	3.86 (2.85, 5.19)	5.84 (4.42, 7.69)	10.43 (8.73, 12.41)	

Mean (95% CI) for the continuous variables: the *p*-value was calculated using the weighted linear regression model. Percentage (95% CI) for the categorical variables: the *p*-value was calculated using a weighted chi-square test. PIR, poverty income ratio; BMI, body mass index; AMD, age-related macular degeneration.

### 3.2 Association between the WWI and AMD

The correlation between the WWI and AMD is presented in Table 2. This study examined the impact of the WWI on AMD prevalence through weighted multivariable logistic regression analyses. While the WWI was considered a consecutive covariate, a significant difference was observed in the association with the presence of AMD in Model 1 compared to the adjusted models (OR: 1.76, 95% CI: 1.52–2.04; OR: 1.12, 95% CI: 0.96–1.32; OR: 1.11, 95% CI: 0.90–1.36). Each unit increase in the WWI increased a participant’s likelihood of having AMD by 76%, as observed in model 1. When the WWI was set as a categorical variable in model 1, the tertile 3 group had a significantly higher risk of developing AMD, 1.9 times greater compared to the lowest tertile group (OR: 2.90, 95% CI: 2.18–3.86). The overall positive trend in this model was also significant, as indicated by the trend test (*p* for trend <0.0001). Nevertheless, this effect remained different in the partially and fully adjusted models (model 2 and model 3). The positive trend in these two models was not significant after adjusting for the covariates included in the study (*p* for trend >0.05). When the WHtR index, representing central obesity, was included, a different association between the WWI and AMD was observed, as shown in Supplementary Table 2.

**Table 2:** Association between the WWI and AMD.

Weight-adjusted-waist index	OR (95%CI) *p*-value					
	Model 1		Model 2		Model 3	
Continuous	1.76 (1.52, 2.04)	<0.0001	1.12 (0.96, 1.32)	0.1695	1.11 (0.90, 1.36)	0.3516
Categories						
Tertile 1	Reference		Reference		Reference	
Tertile 2	1.55 (1.04, 2.30)	0.0390	1.07 (0.70, 1.65)	0.7597	1.10 (0.70, 1.74)	0.6820
Tertile 3	2.90 (2.18, 3.86)	<0.0001	1.28 (0.93, 1.77)	0.1421	1.29 (0.87, 1.89)	0.2369
*p* for trend		<0.0001		0.1112		0.2096

Model 1: No covariates were adjusted. Model 2: Adjusted for sex, age, and ethnicity. Model 3: Adjusted for sex, age, ethnicity, education level, marital status, poverty income ratio (PIR), body mass index (BMI), smoking status, and diabetes and hypertension status. WWI, weight-adjusted-waist index. OR, odd ratio. 95% CI, confidence interval.

**Figure 2:**
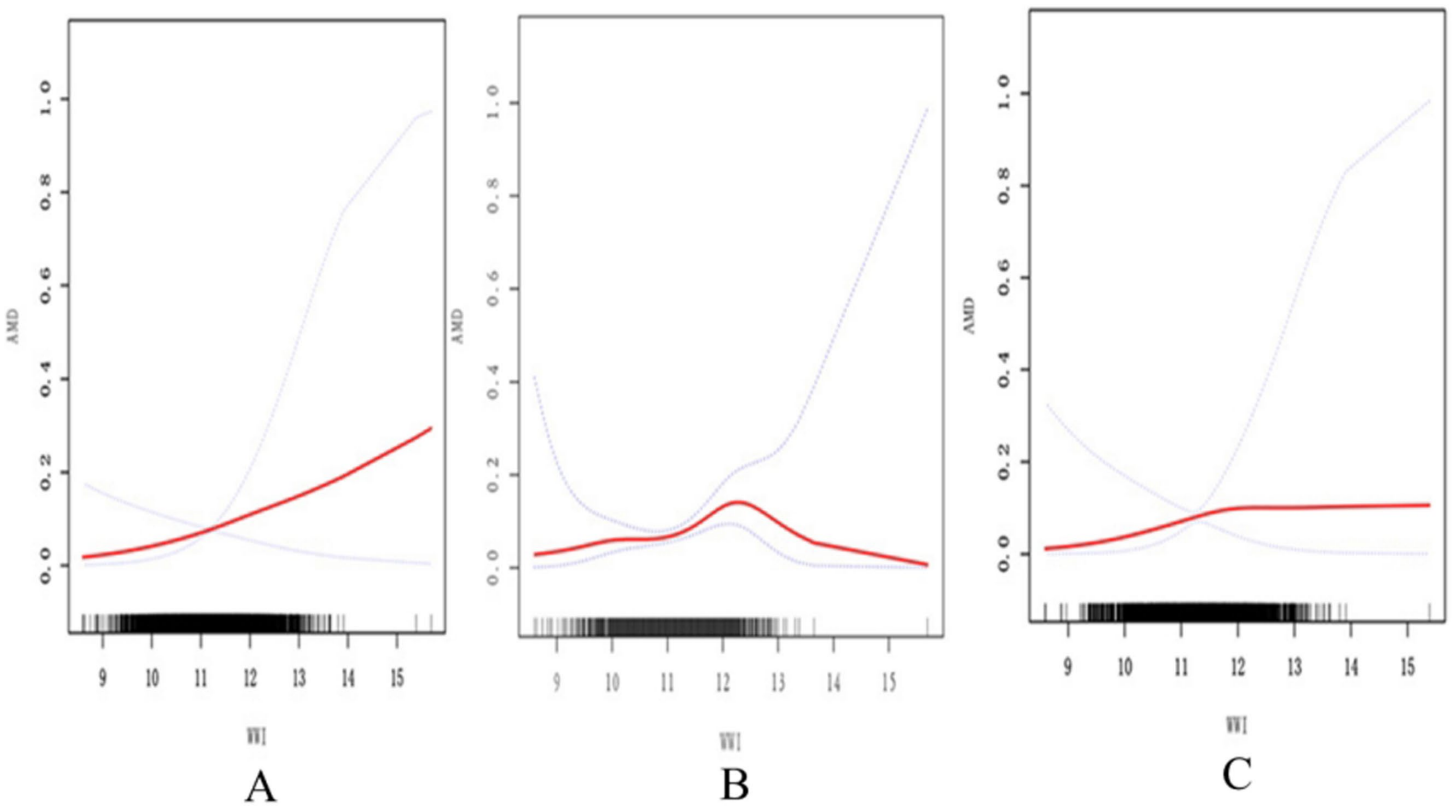
Association between the WWI and AMD assessed using spline smoothing. The black vertical line on the horizontal axis represents the WWI distribution. The smooth curve fit between the variables is shown by the solid red line. The blue bands represent the 95% confidence interval. **(A)** The association between WWI and AMD in the general population. **(B)** The association between WWI and AMD in males. **(C)** The association between WWI and AMD in females All covariates in Table 1 are adjusted. AMD, age-related macular degeneration; WWI, weight-adjusted waist index.

Based on the analyses of spline smoothing, a positive non-linear correlation was observed between the WWI and AMD. A log-likelihood ratio test comparing a single-line (non-segmentation) model and a segmentation regression model in the threshold effect analyses was used to confirm whether a threshold point existed. The threshold effect analyses identified a turning point of 12.11 for the association between the WWI and AMD in the general population (*p* < 0.05) (Figure 2A; Table 3). When the WWI value was below this point, a significant positive increase was observed in the association with AMD (OR 1.50, 95% CI: 1.04–2.16, *p* = 0.0300). When the WWI value exceeded this threshold, there was no statistically significant increase (OR: 0.75, 95% CI: 0.39–1.45, *p* = 0.3943). From a biological perspective, it was hypothesized that this inflection point may represent a critical threshold, beyond which the risk for AMD does not increase significantly, potentially indicating minimal pronounced pathological changes in the retina within this study population. In the subgroups stratified by sex, the WWI was positively associated with AMD in the male participants. Simultaneously, the log-likelihood ratio was below 0.05 in the male participants, indicating a non-linear trend among them (Figure 2B; Table 3). When the value was below 12.2, a significant association with AMD was observed (OR 1.85, 95% CI: 1.22–2.81, *p* = 0.0040). However, above this threshold, this association was not statistically significant (OR 0.32, 95% CI: 0.05–2.08, *p* = 0.2299). For the female participants, the turning point in the non-linear relationship between the WWI and AMD was calculated to be 11.83 (Figure 2C). Below this point, there was a positive association with AMD prevalence (OR: 1.93, 95% CI: 1.28–2.91, *p* = 0.0016), while above this point, the association was not statistically significant (OR: 0.86, 95% CI: 0.47–1.58, *p* = 0.6303), as shown in Table 3.

**Table 3:** Threshold effect analyses of the WWI on AMD.

WWI	OR (95% CI)/ *p-*value
Fitting by standard linear model	1.46 (1.17, 1.82) 0.0009
Fitting by two-piecewise linear model	
Inflection point	12.11
<12.11	1.69 (1.29, 2.22) 0.0001
>12.11	0.85 (0.46, 1.56) 0.5976
Log-likelihood ratio	0.041
Male	
Fitting by standard linear model	1.47 (1.03, 2.09) 0.0314
Fitting by two-piecewise linear model	
Inflection point	12.2
<12.2	1.85 (1.22, 2.81) 0.0040
>12.2	0.32 (0.05, 2.08) 0.2299
Log-likelihood ratio	0.028
Female	
Fitting by standard linear model	1.47 (1.09, 1.98) 0.0114
Fitting by two-piecewise linear model	
Inflection point	11.83
<11.83	1.93 (1.28, 2.91) 0.0016
>11.83	0.86 (0.47, 1.58) 0.6303
Log-likelihood ratio	0.038

Adjusted for sex, age, ethnicity, education level, marital status, poverty income ratio (PIR), body mass index (BMI), smoking, diabetes, and hypertension. WWI, weight-adjusted-waist index. OR, odd ratio. 95% CI, confidence interval.

In addition, considering the WHtR and WC indices, the associations between the WHtR or WC and AMD across the different sex groups were assessed using spline smoothing, as shown in Supplementary Figures 1, 2.

### 3.3 Subgroup analyses

Stratified by multivariable adjustments, subgroup analyses and interaction tests were conducted to further explore potential covariates influencing the association between the WWI and AMD prevalence across the various population groups (Table 4). The results revealed that the correlation between the WWI and AMD remained consistent across all stratified subgroups (all *p* for interaction >0.05). Considering the correlation between the WHtR, WC, and BMI with AMD, additional subgroup analyses are detailed in Supplementary Table 3. The results showed that the associations remained consistent across the specific subgroups based on age and sex (all *p* for interaction >0.05).

**Table 4:** Subgroup analyses of the association between the WWI and AMD.

	OR (95% CI) *p*-value	*p* for interaction
Sex		0.1549
Male	1.40 (0.54, 3.61) 0.4908	
Female	1.74 (0.70, 4.32) 0.2300	
Age (years)		0.6612
40–51	2.03 (0.74, 5.61) 0.1718	
52–64	2.46 (0.94, 6.46) 0.0676	
65–85	2.07 (0.84, 5.12) 0.1150	
Ethnicity		0.5293
Mexican American	1.45 (0.54, 3.94) 0.4626	
Other Hispanic	1.63 (0.58, 4.57) 0.3541	
Non-Hispanic white	1.94 (0.78, 4.81) 0.1520	
Non-Hispanic Black	1.84 (0.68, 4.97) 0.2276	
Other ethnicities	1.01 (0.30, 3.43) 0.9856	
Education level		0.5389
Less than 9th Grade	1.83 (0.71, 4.75) 0.2115	
9-11th grade	1.78 (0.67, 4.70) 0.2457	
High school grad/GED or equivalent	1.54 (0.62, 3.82) 0.3562	
Some college or associate degree	2.08 (0.80, 5.41) 0.1324	
College graduate or above	2.18 (0.83, 5.76) 0.1138	
PIR		0.4738
<1.69	1.72 (0.69, 4.31) 0.2446	
1.69–3.79	1.62 (0.65, 4.01) 0.3004	
>3.79	2.08 (0.81, 5.37) 0.1298	
WC		0.5240
<93.70	1.44 (1.05, 1.99) 0.0248	
93.70–106.40	1.41 (1.00, 2.00) 0.0517	
>106.40	1.15 (0.81, 1.62) 0.4443	
Weight		0.6864
<72.20	1.38 (0.99, 1.94) 0.0599	
72.20–88.20	1.27 (0.86, 1.87) 0.2381	
>88.20	1.17 (0.76, 1.82) 0.4717	
BMI		0.3949
<26.07	2.10 (0.78, 5.66) 0.1409	
26.07–30.91	1.80 (0.61, 5.30) 0.2857	
>30.91	2.41 (0.71, 8.13) 0.1571	
Diabetes		0.6573
Yes	1.98 (0.72, 5.45) 0.1848	
No	1.75 (0.71, 4.29) 0.2231	
Borderline	1.32 (0.39, 4.41) 0.6543	
Smoking		0.8511
Yes	1.69 (0.67, 4.25) 0.2640	
No	1.74 (0.70, 4.30) 0.2308	
Hypertension		0.1384
Yes	2.04 (0.81, 5.14) 0.1311	
No	1.62 (0.66, 3.98) 0.2919	

PIR, poverty income ratio; WC, waist circumference; BMI, body mass index; OR, odd ratio. 95% CI, confidence interval.

### 3.4 ROC analysis

**Figure 3:**
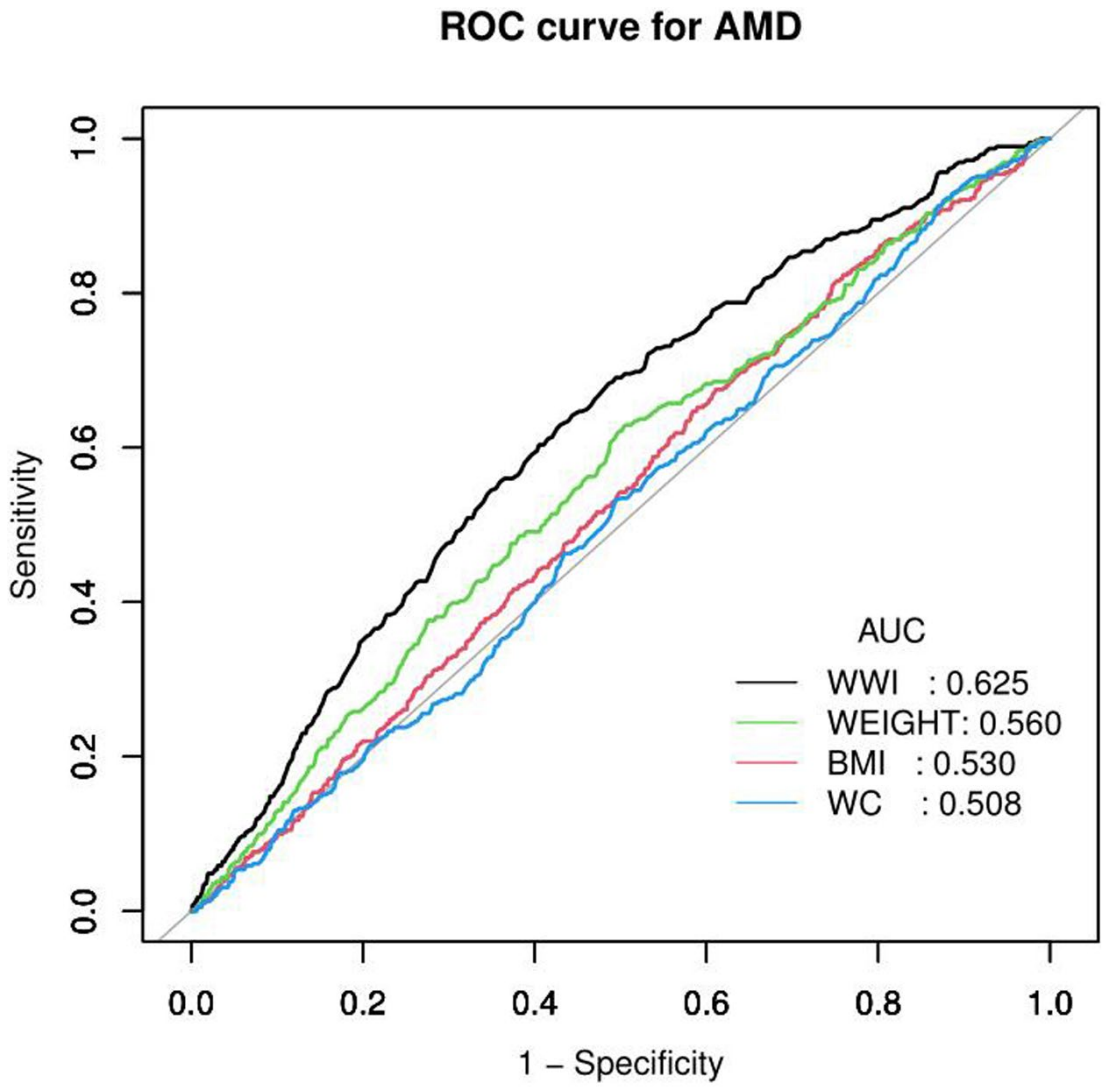
ROC curves between the different anthropometric indices and AMD. ROC, receiver operating characteristic curve; AUC, area under the curve; AMD, age-related macular degeneration; WWI, weight-adjusted waist index; BMI, Body mass index; WC, waist circumference.

Through receiver operating characteristic (ROC) curve analysis, the predictive power of the WWI for AMD was tested and compared with other obesity assessment indicators. For AMD incidence, the corresponding area under the curve (AUC) values in the model were as follows: WWI, 0.625; weight, 0.560; BMI, 0.530; and WC, 0.508. The results indicated that the WWI had a greater capability to identify AMD risk compared to BMI, WC, and weight (Figure 3). In examining the WHtR index, the ROC curves are depicted in Supplementary Figure 3. The AUC value for the WHtR shown in this Supplementary Figure 3 was 0.537, which was lower than the value for the WWI. This suggests that the WWI may be regarded as a more effective and valuable obesity indicator for AMD compared to other central obesity indices.

## 4 Discussion

This cross-sectional study showed that the WWI had a significant positive non-linear relationship with AMD incidence. The subgroup analyses by sex revealed consistent non-linear connections between the WWI and AMD in both groups.

As the global prevalence of obesity and the incidence of obesity-related diseases rise, the associated economic burden on healthcare continues to increase (32). The degree of obesity is currently evaluated using an increasing number of new indicators. Traditional anthropometric methods used to measure obesity, including BMI and WC, while convenient to obtain, have difficulty distinguishing between muscle mass and adipose tissue (42). Consequently, numerous studies related to BMI have theorized the “obesity paradox” (43, 44). This paradox suggests that individuals who are obese or overweight may have better prognoses than the average-weight population to some extent (45). This is in line with a population-based study in Korea, which found that non-overweight status, often regarded as a positive health indicator, was detrimentally associated with AMD (46). These inverse associations with obesity, known as the obesity paradox, have also been observed in other diseases (47). A population-based study conducted on diabetic patients in Taiwan indicated that this observed phenomenon was predominantly associated with non-cancer mortality, implying a survival advantage among obese individuals (48). Several plausible explanations are offered for these adverse correlations. First, the aging process tends to result in a selective group of non-obese individuals, given that mortality rates are higher among obese individuals compared to non-obese populations (49). Secondly, ethnic variations may mediate the correlation between obesity and AMD, as previously noted (50, 51). In addition, a study involving white individuals found that the relationship between the two differed by sex (52).

Gradually, a new obesity index called the WWI was introduced in 2018 and was deemed to more accurately reflect the morbidity and mortality associated with obese-related diseases (53). For instance, Kim et al. (36) explored a positive relationship between the WWI and belly fat accumulation and a negative relationship between the WWI and abdominal muscle mass, which were not affected by ethnicity. While body weight has always reflected fat distribution and adiposity in adults due to the stability of height, many studies have shown that central obesity, especially fat accumulation in deep subcutaneous tissues, is a more representative indicator. The WWI, which combines weight and WC, serves as an integrated index reflecting body composition and better representing physical conditions. A high WWI often indicates that individuals with a larger WC have greater fat mass and proportionally lower muscle mass (54). In addition, inflammatory reactions and metabolic disorders are mostly caused by central obesity (55, 56). In the context of assessing abdominal obesity and associated health risks, WC has been identified as a significant predictor of cardiometabolic risk in previous studies (57). In Colombian older adults, WC demonstrated moderate discriminative ability in detecting type 2 diabetes mellitus in the elderly population (58). Interventions aimed at reducing central obesity, as measured by WC, may be more effective in preventing severe metabolic disorders, including myocardial infarction (59). The use of the WHtR for the detection of abdominal obesity and its associated health risks was first proposed in the mid-1990s (60). A multi-ethnic study indicated that the WHtR is superior to both WC and BMI in identifying cardiometabolic risk factors such as hypertension and diabetes (61). However, similar to BMI, both the WHtR and WC are limited in their ability to differentiate and quantify the proportions of muscle and fat in the human body, and individual differences exist greatly. A recent study has increasingly demonstrated that the WWI is a more promising indicator than WC, BMI, and the WHtR for diagnosing diabetes and cardiovascular disease related to adiposity (62). In brief, it is credible to use the WWI as a better predictive tool for obesity (32).

The study demonstrated that the WWI was positively associated with AMD prevalence after adjusting for all confounders. This result aligns with those of previous studies, such as the Age-Related Eye Disease Study (AREDS), which found that larger weight gains were related to a higher risk of AMD (63). One possible reason is that excessive caloric intake increases the risk of AMD, as it may induce oxidative stress reactions in the eyes, damaging the retina (64). Similarly, obesity was found to be a pro-inflammatory state, contributing to the chronic inflammatory response of AMD (65). Previous studies have confirmed that both generalized and abdominal obesity are associated with AMD (66). Zhang et al. (67) summarized prospective studies on the correlation between obesity and AMD. They elaborated that overweight may be a potential inducement for the onset of AMD. In some studies, the secretion of pro-inflammatory stimuli, such as monocyte chemoattractant protein-1 and tumor necrosis factor-*α*, was significantly elevated in obesity groups, triggering monocyte migration and infiltration, which led to retinal pigment epithelial dysfunction that produced AMD lesions (68, 69). At the molecular level of the WWI-AMD link, various classes of lipid and lipoprotein metabolism have been implicated in the pathogenesis of AMD (70). Given that multiple cholesterol-related genes serve as risk factors for AMD, the cholesterol-AMD link is robust, supported by evidence such as elevated cholesterol content in drusen, the aging of Bruch’s membrane, and the recent identification of subretinal lesions (71).

In contrast to previous studies, this present study used the WWI as a marker to assess the correlation between obesity and AMD, investigating the association between the WWI and AMD across different populations by subgroups. Specifically, the WWI-AMD link revealed the same positive non-linear relationship in both sex groups. Generally, female individuals tend to store fat primarily in peripheral areas, such as the subcutaneous tissues of the buttocks, thighs, and limbs, giving them a pear-shaped body. In contrast, obese male individuals typically accumulate more visceral fat (72). Unlike what is typically observed, this study indicated that the female individuals were as susceptible to central obesity as the male individuals, which aligns with findings from some other studies (73–75). The causes of this discrepancy may be related to the factors listed below. Early evidence showed that female individuals had a higher mean WWI than male individuals (54). This suggested that female individuals had a worse body composition than men, contributing to this positive association. In addition, individual differences in this survey may have influenced the results, particularly since the female population exhibited a higher proportion of central fat, similar to the male group. Furthermore, female individuals may be more focused on diet and external factors, although individual variations in estrogen and hormone metabolism could offer protective benefits.

There are several advantages to this study. First, all the data incorporated were derived from the NHANES database, which has a large sample size and represents international diversity. In addition, it is the first cross-sectional study to examine the relationship between the WWI and AMD. However, there are some limitations as well. First, a definite causal association between the WWI and AMD was difficult to establish given the cross-sectional nature of the study. Second, since this data survey was conducted among the United States population, it remains uncertain whether similar conclusions can be drawn for other countries and ethnicities. Third, although many important covariates were adjusted for, there is no guarantee that residual variables not included would have had an impact on the results. Finally, due to the limitations of AMD’s investigable scope, relatively old data had to be utilized, which limited its timeliness and impacted the current trends.

## 5 Conclusion

In summary, higher WWI was associated with an increased prevalence of AMD in this study. The research underscores the potential of the WWI as a reliable predictive biomarker for AMD, with spline smoothing analyses confirming a non-linear positive relationship between the WWI and AMD prevalence. These findings emphasize the significance of early prediction and intervention for AMD in the general population. Nonetheless, future research should prioritize investigating diverse racial groups, conducting longitudinal studies to establish cause-and-effect relationships, and potentially utilizing intelligent ophthalmology (IO) technology to explore molecular mechanisms in greater depth, as the pathophysiology of AMD warrants further exploration (76).

## Data Availability

The datasets presented in this study can be found in online repositories. The names of the repository/repositories and accession number(s) can be found at: https://www.cdc.gov/nchs/nhanes/.

## Footnotes

^1^ http://www.r-project.org
