# Supplementary material for: Association between the weight-adjusted waist index and age-related macular degeneration in US adults aged≥40 years: the NHANES 2005–2008
Source: Front Med (Lausanne). 2025 Mar 6;12:1552978. doi: 10.3389/fmed.2025.1552978 (PMC11922941; doi:10.3389/fmed.2025.1552978)
Supplement: Supplementary file 1 [file Supplementary_file_1.pdf]

Exposure variable :

WHtR (waist-to-height ratio) was calculated as below:

WHtR=waist circumference (cm)÷（height）(cm)

Supplementary Figure 1

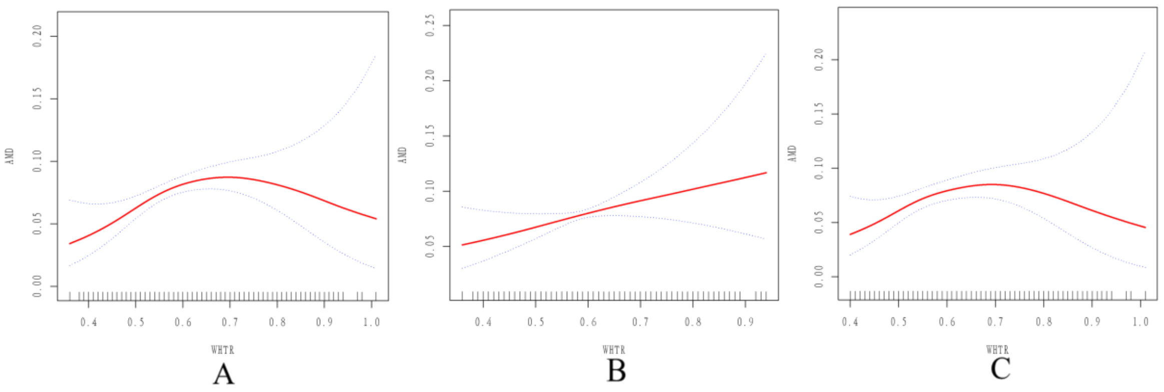

Figure 1. Association between WHtR and AMD by spline smoothing.

The black vertical line on the horizontal axis represents the WHtR distribution. Smooth curve fit between variables is showed by solid red line. Blue bands represent 95% confidence interval. Figure 1A represents the association between WHtR and AMD in the general population. Figure 1B represents the association between WHtR and AMD in males. Figure 1C represents the association between WHtR and AMD in females. Abbreviations: AMD, age-related macular degeneration; WHtR, waist-to-height ratio.

Supplementary Figure 2

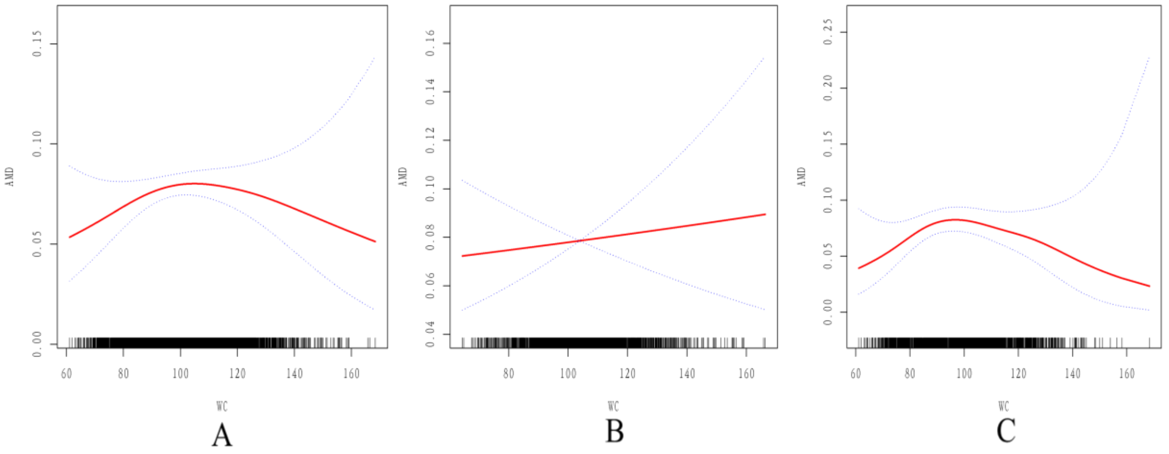

Figure 2. Association between WC and AMD by spline smoothing.

The black vertical line on the horizontal axis represents the WC distribution. Smooth curve fit between variables is showed by solid red line. Blue bands represent 95% confidence interval. Figure 2A represents the association between WC and AMD in the general population. Figure 2B represents the association between WC and AMD in males. Figure 2C represents the association between WC and AMD in females. Abbreviations: AMD, age-related macular degeneration; WC, waist circumference.

Supplementary Figure 3

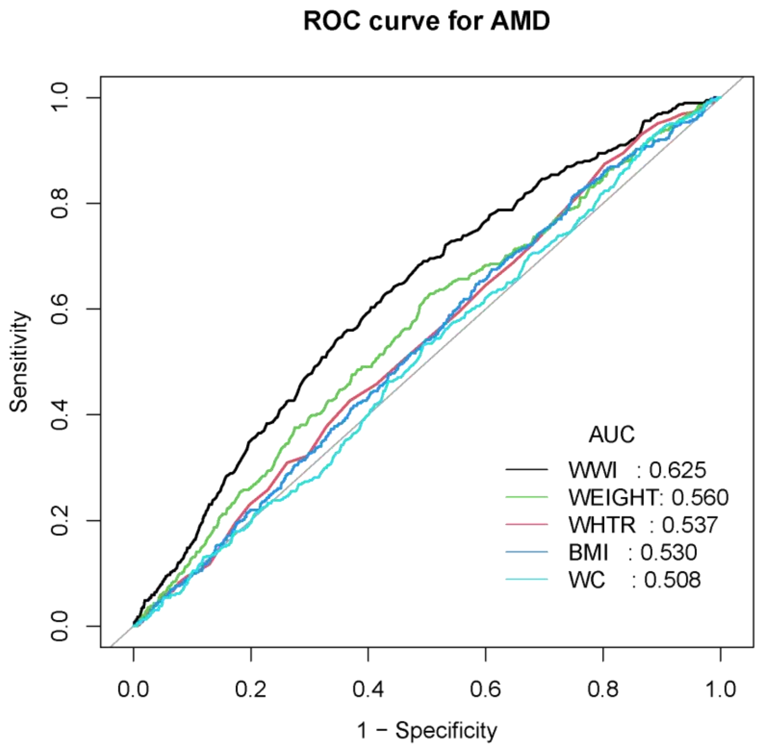

Figure 3. ROC curves between different anthropometric indices and AMD

Abbreviations: ROC, receiver operating characteristic curve; AUC, area under curve; AMD, age-related macular degeneration; WWI, weight-adjusted waist index; WHtR,waist-to-height ratio; BMI, body mass index; WC, waist circumference.

Supplementary Table 1 (after adding to WHtR.)

Table 1 Baseline characteristics of the study population

Weight-adjusted waist index	Tertile 1 N=1711	Tertile 2 N=1697	Tertile 3 N=1724	*P*-value
Gender (%)				<0.0001
Male	48.14 (44.82 ,51.47)	51.37 (48.35 ,54.39)	40.42 (38.21 ,42.67)	
Female	51.86 (48.53 ,55.18)	48.63 (45.61 ,51.65)	59.58 (57.33 ,61.79)	
Age (years)	52.04 (51.26 ,52.83)	56.18 (55.34 ,57.02)	62.25 (61.30 ,63.21)	<0.0001
Race (%)				<0.0001
Mexican Americans	2.81 (2.12 ,3.71)	5.17 (3.71 ,7.16)	6.09 (3.95 ,9.29)	
Other Hispanic	2.48 (1.50 ,4.09)	2.43 (1.56 ,3.78)	4.27 (2.84 ,6.39)	
Non-Hispanic white persons	77.73 (73.45 ,81.49)	78.67 (73.95 ,82.73)	77.96 (72.38 ,82.69)	
Non-Hispanic Black	12.41 (9.55 ,15.96)	8.32 (6.11 ,11.23)	7.54 (5.53 ,10.21)	
Other races	4.58 (3.04 ,6.83)	5.41 (3.98 ,7.33)	4.12 (2.71 ,6.22)	
Education level (%)				<0.0001
Less than 9th Grade	3.27 (2.32 ,4.60)	6.05 (4.35 ,8.38)	10.13 (8.06 ,12.65)	
9-11th grade	9.11 (7.19 ,11.47)	10.20 (8.02 ,12.90)	14.63 (12.56 ,16.96)	
High school grad/GED or equivalent	22.43 (20.07 ,24.97)	28.85 (25.63 ,32.29)	29.49 (27.26 ,31.82)	
Some college or associate degree	29.38 (26.52 ,32.43)	28.27 (24.79 ,32.04)	26.98 (23.17 ,31.17)	
College graduate or above	35.81 (31.42 ,40.45)	26.62 (22.88 ,30.74)	18.78 (15.68 ,22.32)	
Marital status (%)				<0.0001
Married	65.80 (62.00 ,69.41)	68.37 (64.56 ,71.95)	60.76 (58.01 ,63.44)	
Widowed	4.03 (3.08 ,5.26)	7.53 (6.28 ,9.00)	14.46 (12.26 ,16.97)	
Divorced	15.58 (13.08 ,18.46)	12.80 (10.65 ,15.31)	13.20 (10.90 ,15.89)	
Separated	2.37 (1.54 ,3.62)	3.04 (2.07 ,4.44)	1.60 (1.11 ,2.31)	
Never married	7.01 (5.31 ,9.20)	4.83 (3.63 ,6.39)	6.06 (4.87 ,7.53)	
Living with partner	5.21(4.16,6.51)	3.44(2.42,4.86)	3.92(2.66,5.73)	
PIR	3.62 (3.48 ,3.75)	3.38 (3.22 ,3.53)	2.90 (2.75 ,3.04)	<0.0001
Waist	90.02 (89.22 ,90.82)	102.58	112.44	<0.0001

circumference (cm)		(101.90 ,103.26)	(111.47 ,113.42)	
Weight (kg)	76.70 (75.42 ,77.98)	85.27 (84.15 ,86.38)	89.08 (87.56 ,90.60)	<0.0001
Height(cm)	170.85(170.17,171.52)	169.41(168.91,169.91)	164.76(164.24,165.28)	<0.0001
BMI (kg/m^2)	26.14 (25.78 ,26.51)	29.56 (29.27 ,29.85)	32.59 (32.11 ,33.06)	<0.0001
WHtR	0.53(0.52,0.53)	0.61(0.60,0.61)	0.68(0.68,0.69)	<0.0001
Diabetes (%)				<0.0001
Yes	3.52 (2.80 ,4.42)	9.53 (7.35 ,12.26)	20.91 (18.49 ,23.56)	
No	95.55 (94.29 ,96.53)	88.92 (85.89 ,91.37)	75.48 (72.45 ,78.27)	
Borderline	0.93 (0.51 ,1.69)	1.55 (0.92 ,2.60)	3.61 (2.59 ,5.00)	
Smoking (%)				0.0004
Yes	47.25 (43.63 ,50.91)	53.50 (49.84 ,57.13)	55.18 (52.25 ,58.07)	
No	52.75 (49.09 ,56.37)	46.50 (42.87 ,50.16)	44.82 (41.93 ,47.75)	
Hypertension (%)				<0.0001
Yes	28.71 (25.93 ,31.66)	43.10 (39.42 ,46.85)	57.42 (54.58 ,60.20)	
No	71.29 (68.34 ,74.07)	56.90 (53.15 ,60.58)	42.58 (39.80 ,45.42)	
AMD (%)				<0.0001
No	96.14 (94.81 ,97.15)	94.16 (92.31 ,95.58)	89.57 (87.59 ,91.27)	
Yes	3.86 (2.85 ,5.19)	5.84 (4.42 ,7.69)	10.43 (8.73 ,12.41)	

Mean (95% CI) for continuous variables: the *P*-value was calculated by the weighted linear regression model. Percentage (95% CI) for categorical variables: the *P*-value was calculated by the weighted chi-square test. Abbreviations: PIR, poverty income ratio; BMI, body mass index; WHtR, waist-to-height ratio; AMD, age-related macular degeneration.

Supplementary Table 2 (after adding to WHtR.)

Table 2 Association between WWI and AMD

Weight-adjusted-waist index	OR (95%CI) P-value					
	Model1		Model 2		Model 3	
Continuous	1.76 (1.52, 2.04)	<0.0001	1.12 (0.96, 1.32)	0.1695	1.19 (0.45, 3.11)	0.7325
Categories						
Tertile1	Reference		Reference		Reference	
Tertile2	1.55 (1.04, 2.30)	0.0390	1.07(0.70, 1.65)	0.7597	1.09(0.65, 1.83)	0.7458
Tertile3	2.90(2.18, 3.86)	<0.0001	1.28 (0.93, 1.77)	0.1421	1.32(0.77, 2.28)	0.3489
p for trend		<0.0001		0.1112		0.3388

Model 1: no covariates were adjusted.

Model 2: adjusted for gender, age, and race.

Model 3: adjusted for gender, age, race, education level, marital status, poverty income ratio (PIR), body mass index (BMI), waist-to-height ratio(WHtR), smoking status, diabetes and hypertension status.

Abbreviations: WWI, weight-adjusted-waist index. AMD, age-related macular degeneration. OR, odd ratio. 95% CI, confidence interval.

Supplementary Table 3 (conducted for the cutoffs of age and sex)

Table 3 Subgroup analyses of the association between obesity indices and AMD

Variables	WHtR and AMD	WC and AMD	BMI and AMD
	OR (95% CI) *P*-value	OR (95% CI) *P*-value	OR (95% CI) *P*-value
Gender			
Male	4.30(1.02,5.19)0.1042	0.87(0.73,1.05)0.1528	0.88(0.61,1.25)0.4650
Female	4.17(2.02, 6.34)0.0932	0.88(0.72,1.06)0.1741	0.86(0.62,1.19)0.3637
P for interaction	0.9865	0.8507	0.5231
Age(years)			
40-51	1.33(1.02,2.96)0.1081	0.88(0.73,1.06)0.1723	0.82(0.62,1.10)0.1889
52-64	5.15(3.02,9.42)0.0869	0.88(0.74,1.06)0.1811	0.84(0.63,1.12)0.2415
65-85	1.26(1.02,1.49)0.1009	0.87(0.73,1.04)0.1355	0.83(0.62,1.11)0.2104
P for interaction	0.6522	0.3396	0.7129

Abbreviations: WHtR, waist-to-height ratio; WC, waist circumference; BMI, body mass index; AMD, age-related macular degeneration；OR, odd ratio；95% CI, confidence interval.

Supplementary Table 4 (conducted for males)

Table 4 Baseline characteristics of the study population

Characteristic	For male		*P*-value
	No AMD (n=4741)	With AMD (n=391)	
Age(years)	55.14 (54.44 ,55.84)	64.91(62.51 ,67.31)	<0.0001
Race(%)			0.1073
Mexican Americans	4.75 (3.41 ,6.59)	4.51 (2.52 ,7.95)	
Other Hispanic	2.88 (1.93 ,4.27)	3.16 (1.53 ,6.41)	
Non-Hispanic white persons	77.97 (73.46 ,81.90)	82.72(75.36 ,88.23)	
Non-Hispanic Black	9.63 (7.53 ,12.24)	4.23 (2.22 ,7.91)	
Other races	4.77 (3.41 ,6.65)	5.37 (2.30 ,12.05)	
Education level (%)			0.8252
Less than 9th Grade	6.78 (5.30 ,8.64)	9.18 (4.92 ,16.47)	
9-11th grade	10.65 (8.71 ,12.96)	10.70 (6.34 ,17.51)	
High school grad/GED or Equivalent	25.45 (22.66 ,28.45)	26.54(19.13 ,35.55)	
Some college or associate degree	27.63 (24.91 ,30.53)	27.56(18.97 ,38.21)	
College graduate or above	29.49 (25.46 ,33.86)	26.02(17.32 ,37.14)	
Marital Status (%)			0.0678
Married	71.33 (68.39 ,74.09)	73.41(64.12 ,81.01)	
Widowed	2.91 (2.32 ,3.65)	5.72 (2.97 ,10.73)	
Divorced	12.17 (10.31 ,14.32)	11.14 (6.09 ,19.50)	
Separated	2.08 (1.27 ,3.40)	3.86 (2.02 ,7.24)	
Never married	6.45 (5.10 ,8.12)	3.32 (1.81 ,6.01)	
Living with partner	5.06 (3.91 ,6.52)	2.55 (0.89 ,7.11)	
PIR	3.33 (3.18 ,3.49)	2.90 (2.56 ,3.24)	0.0234
Waist circumference (cm)	104.64(103.71 ,105.56)	104.96(102.72 ,107.20)	0.7864
Weight(kg)	90.81 (89.71 ,91.92)	87.24 (84.09 ,90.39)	0.0359
Height(cm)	176.28(175.82 ,176.75)	174.09(172.72 ,175.47)	0.0036
WHtR	0.59 (0.59 ,0.60)	0.60 (0.59 ,0.62)	0.1650
BMI (kg/m^2)	29.16 (28.84 ,29.49)	28.69 (27.86 ,29.51)	0.2823
WWI	11.01 (10.97 ,11.06)	11.28 (11.15 ,11.40)	0.0003
Diabetes (%)			0.1495
Yes	10.01 (8.63 ,11.59)	13.65 (9.04 ,20.11)	
No	87.69 (85.71 ,89.43)	82.93 (75.93 ,88.21)	
Borderline	2.30 (1.69 ,3.13)	3.41 (1.88 ,6.12)	
Smoking (%)			0.1970
Yes	58.76 (55.57 ,61.87)	64.32 (55.21 ,72.49)	
No	41.24 (38.13 ,44.43)	35.68 (27.51 ,44.79)	
Hypertension (%)			0.0717
Yes	39.35 (36.47 ,42.31)	48.67 (38.41 ,59.05)	
No	60.65 (57.69 ,63.53)	51.33 (40.95 ,61.59)	

Mean (95% CI) for continuous variables: the *P*-value was calculated by the weighted linear

regression model. Percentage (95% CI) for categorical variables: the *P*-value was calculated by the weighted chi-square test. Abbreviations: PIR, poverty income ratio; BMI, body mass index; WHtR, waist-to-height ratio; WWI, weight-adjusted waist index; AMD, age-related macular degeneration.

Supplementary Table 5 (conducted for females)

Table 5 Baseline characteristics of the study population

Characteristic	For female No AMD (n=4741)	For female With AMD (n=391)	*P*-value
Age(years)	55.78 (54.95 ,56.61)	69.08 (66.56 ,71.60)	<0.0001
Race(%)			<0.0001
Mexican Americans	4.36 (3.25 ,5.82)	3.24 (1.91 ,5.44)	
Other Hispanic	3.10 (1.99 ,4.79)	1.57 (0.52 ,4.62)	
Non-Hispanic white persons	77.15 (72.48 ,81.22)	90.19 (85.15 ,93.65)	
Non-Hispanic Black	10.50 (7.85 ,13.92)	3.51 (1.81 ,6.70)	
Other races	4.89 (3.70 ,6.45)	1.48 (0.33 ,6.38)	
Education level (%)			0.0876
Less than 9th Grade	4.98 (3.92 ,6.30)	10.20 (5.13 ,19.26)	
9-11th grade	11.05 (9.13 ,13.31)	14.25 (8.08 ,23.91)	
High school grad/GED or Equivalent	27.34 (25.15 ,29.64)	28.59 (20.59 ,38.20)	
Some college or associate degree	29.13 (26.77 ,31.62)	27.18 (19.15 ,37.04)	
College graduate or above	27.50 (24.33 ,30.92)	19.78 (12.50 ,29.85)	
Marital Status (%)			<0.0001
Married	60.73 (57.63 ,63.75)	46.51 (37.14 ,56.14)	
Widowed	10.83 (9.56 ,12.25)	36.47 (30.26 ,43.18)	
Divorced	15.77 (13.58 ,18.24)	14.11 (9.00 ,21.43)	
Separated	2.71 (1.98 ,3.70)	0.43 (0.12 ,1.53)	
Never married	6.13 (4.72 ,7.92)	0.86 (0.29 ,2.55)	
Living with partner	3.82 (3.09 ,4.71)	1.61 (0.38 ,6.64)	
PIR	3.16 (3.03 ,3.29)	2.60 (2.30 ,2.90)	0.0014
Waist circumference (cm)	96.48 (95.56 ,97.40)	97.12 (94.68 ,99.56)	0.6377
Weight(kg)	76.43 (75.40 ,77.46)	72.24 (69.18 ,75.30)	0.0188
Height(cm)	162.24(161.87 ,162.61)	159.67(158.70 ,160.63)	0.0001
WHtR	0.60 (0.59 ,0.60)	0.61 (0.59 ,0.63)	0.1059
BMI (kg/m^2)	29.01 (28.65 ,29.38)	28.35 (27.16 ,29.53)	0.3107
WWI	11.09 (11.04 ,11.14)	11.48 (11.36 ,11.59)	<0.0001
Diabetes (%)			0.7654
Yes	10.23 (8.32 ,12.51)	11.26 (7.73 ,16.12)	
No	88.37 (85.87 ,90.47)	87.08 (82.50 ,90.60)	
Borderline	1.41 (0.96 ,2.06)	1.66 (0.75 ,3.63)	
Smoking (%)			0.2344
Yes	44.43 (41.09 ,47.83)	49.53 (41.92 ,57.16)	
No	55.57 (52.17 ,58.91)	50.47 (42.84 ,58.08)	
Hypertension (%)			0.0254
Yes	41.93 (38.86 ,45.06)	53.41 (43.06 ,63.47)	
No	58.07 (54.94 ,61.14)	46.59 (36.53 ,56.94)	

Mean (95% CI) for continuous variables: the *P*-value was calculated by the weighted linear

regression model. Percentage (95% CI) for categorical variables: the *P*-value was calculated by the weighted chi-square test. Abbreviations: PIR, poverty income ratio; BMI, body mass index; WHtR, waist-to-height ratio; WWI, weight-adjusted waist index; AMD, age-related macular degeneration.
